# Patient Portal Use Among Family Caregivers of Individuals With Dementia and Cancer: Regression Analysis From the National Study of Caregiving

**DOI:** 10.2196/44166

**Published:** 2023-12-20

**Authors:** Reed W R Bratches, Jaclyn A Wall, Frank Puga, Giovanna Pilonieta, Rita Jablonski, Marie Bakitas, David S Geldmacher, J Nicholas Odom

**Affiliations:** 1 School of Nursing, University of Alabama at Birmingham, Birmingham, AL, United States; 2 Department of Neurology, University of Alabama at Birmingham, Birmingham, AL, United States

**Keywords:** patient portal, palliative care, family caregiver, caregiver, dementia, cancer, clinic, age, race, gender, employment, education, model, ethnicity, health system, intervention, regression analysis

## Abstract

**Background:**

Family caregivers are often inexperienced and require information from clinic visits to effectively provide care for patients. Despite reported deficiencies, 68% of health systems facilitate sharing information with family caregivers through the patient portal. The patient portal is especially critical in the context of serious illnesses, like advanced cancer and dementia, where caregiving is intense and informational needs change over the trajectory of disease progression.

**Objective:**

The objective of our study was to analyze a large, nationally representative sample of family caregivers from the National Study of Caregiving (NSOC) to determine individual characteristics and demographic factors associated with patient portal use among family caregivers of persons living with dementia and those living with cancer.

**Methods:**

We conducted a secondary data analysis using data from the 2020 NSOC sample of family caregivers linked to National Health and Aging Trends Study. Weighted regression analysis by condition (ie, dementia or cancer) was used to examine associations between family caregiver use of the patient portal and demographic variables, including age, race or ethnicity, gender, employment status, caregiver health, education, and religiosity.

**Results:**

A total of 462 participants (representing 4,589,844 weighted responses) were included in our analysis. In the fully adjusted regression model for caregivers of persons living with dementia, Hispanic ethnicity was associated with higher odds of patient portal use (OR: 2.81, 95% CI 1.05-7.57; *P*=.04), whereas qualification lower than a college degree was associated with lower odds of patient portal use by family caregiver (OR 0.36, 95% CI 0.18-0.71; *P*<.001. In the fully adjusted regression model for caregivers of persons living with cancer, no variables were found to be statistically significantly associated with patient portal use at the .05 level.

**Conclusions:**

In our analysis of NSOC survey data, we found differences between how dementia and cancer caregivers access the patient portal. As the patient portal is a common method of connecting caregivers with information from clinic visits, future research should focus on understanding how the portal is used by the groups we have identified, and why.

## Introduction

Family caregivers include spouses, partners, other family members, and close friends, who provide uncompensated assistance to individuals with illness or disability in managing their health care [1]. Family caregivers are predominantly female and spend approximately 3 hours per day on caregiving tasks, though more advanced conditions such as cancer and dementia require more caregiving hours daily [1]. Family caregivers often lack formal training and rely on credible external information sources to perform their tasks competently and deliver effective care to their care recipients [2]. The need for information has increased in recent years primarily due to patients’ preference for receiving care within their homes, rather than within nursing homes or other staffed care facilities. This shift has led to family caregivers requiring more, and increasingly specific, information from health care systems [3,4].

The informational needs of family caregivers are particularly elevated in the context of patients receiving palliative care. Caregivers have reported the need for understanding medication side effects, disease progression, and pain management strategies [5]. As conditions progress, caregivers are often asked to provide increasingly complex care, which may involve managing multiple medications, administering complex injections, and coordinating care among multiple health care providers [6-8]. Caregivers of individuals with complex needs express concerns regarding the timing, dosage, and proper administration of medications [5].

Currently, patients with cancer and dementia constitute approximately 35% of those receiving palliative care [9]. By 2040, the number of patients with cancer using palliative care services is projected to increase by 45%, whereas the number of patients with advanced dementia using palliative care services is projected to increase 370% [10]. Caregivers of patients with cancer and dementia report experiencing higher levels of burden, emotional stress, and psychological distress than other caregivers; they also tend to provide assistance with a similarly high number of caregiving tasks when compared to other caregivers [11]. Although there are important differences in the nature of their caregiving tasks, such as acute care associated with cancer treatments provided by caregivers of individuals with cancer, and the prolonged cognitive challenges faced by caregivers of individuals with dementia, both caregiver groups deliver complex care, resulting in increased informational needs [12]. The informational needs of caregivers are particularly heightened when the individuals they care for are approaching the end of life, since conditions may change frequently, and caregivers may find themselves less confident in their caregiving responsibilities [13].

Electronic patient portals, which are online health record systems that facilitate messaging and information exchange among patients, caregivers, and health care providers, can be helpful for caregivers, especially those with high-intensity caregiving situations such as cancer and dementia [14].

A systematic review of studies focusing on family caregivers with information from clinic visits identified the patient portal as a primary pathway for engaging family caregivers [15]. However, little is known about the demographic factors associated with family caregiver use of the patient portal to augment their caregiving responsibilities. A study comprising 3026 family caregivers found that 49% of the caregivers thought that the patient portal could be helpful for their caregiving duties; however, the authors did not inquire about their use of the patient portal [16].

Health systems have recognized patient portals as a primary means of engaging with clinic visit information. These portals represent a vital source of information for family caregivers, especially for caregivers of individuals living with dementia [17]. Although individuals with dementia are just as likely to be registered for patient portals as those without dementia, those with dementia are three times more likely to have a family caregiver who accesses their portal and actively engages with the portal’s information [18]. Qualitative work in regional health systems have found that caregivers with lower health literacy are more likely to face navigational barriers when accessing patient portals [19,20]. However, it remains uncertain how other demographic characteristics of caregivers assisting high-need patients may be associated with patient portal use. A recent cohort study of 49,382 patients from Johns Hopkins Health System concluded that there is a critical need to better support patients and caregivers through patient portals [18]. It is important to discern which groups may benefit the most from targeted interventions and support.

The objective of our study was to analyze a large, nationally representative sample of family caregivers from NSOC to determine the characteristics of family caregivers of individuals with dementia and cancer and the demographic factors associated with their use of patient portals. Understanding these factors will allow researchers and health systems to better comprehend disparities in usage and develop strategies and interventions to help improve access and use of patient portals by family caregivers. We hypothesized that caregivers of different demographic characteristics would access the patient portal at different rates.

## Methods

### Data Source

This secondary data analysis used data from NSOC Round IV, conducted in 2021. NSOC surveyed 1938 family caregivers identified by Medicare beneficiaries who were aged 65 years or older and had participated in Round XI of the 2020 National Health and Aging Trends Study (NHATS), an annual health-related nationally representative study designed for individuals aged 65 years and older in the United States. NHATS collects detailed information on participants’ physical and cognitive capacity, demographic features, and living situations [21]. NSOC does not specifically identify caregivers of individuals with dementia or cancer. However, we derived this information by linking the restricted NSOC files with NHATS by using the anonymous patient identification number and using patient-level information of the presence or absence of dementia and cancer to determine whether their linked caregiver provided care for a patient with cancer or dementia. The reporting of this study complies with the Reporting of Studies Conducted Using Observational Routinely-collected Data (RECORD) Statement (Checklist 1).

### Inclusion Criteria

For inclusion in our analysis, participants in NSOC must have provided care for an individual person in NHATS, as defined by matching the NSOC and NHATS sampling person ID variable in the survey year, regardless of whether the NHATS participant was alive at the end of the survey year. NSOC participants must provide care to an individual with either cancer or dementia, as defined in the NHATS “CONDITION” variable.

### Relevant Measures

Covariates were selected a priori by the study team based on their clinical expertise and knowledge, including race or ethnicity, age, employment status, education, caregiver health, and religiosity. We used precoded NSOC demographic variables for race, ethnicity, and age. For employment status, we considered a caregiver employed if they currently worked for payment. We categorized the level of education based on whether a caregiver had completed college or whether they had a college degree or higher. Caregiver health and religiosity were dichotomized as good or poor and religious or nonreligious, respectively, based on the Press-Ganey Top Box Scoring system [22].

For our outcome measure, we considered a caregiver as a user of the patient portal in a caregiving capacity when they responded “yes” to using online patient portals to access information about the patient. Dementia and cancer were dichotomized as “yes” or “no” based on the presence and absence of either condition in NHATS. The severity or stage of condition was not collected in NHATS and, therefore, it was not accounted for in our analysis. Although participants could select both cancer and dementia, we excluded those with both cancer and dementia due to small cell sizes per the NHATS/NSOC analytic guidelines [23].

### Analytic Strategy

We conducted weighted logistic regression analysis on our outcome measure and variables of interest. We established a predefined alpha level of .05 to determine statistical significance.

We used the NSOC-provided analytical survey weights and included domain variables for the presence of cancer or dementia in the relevant analyses to preserve the effects of the sampling weights in our analysis. Of note, this analysis only included family caregivers who provided care to individuals residing in community or non-nursing home settings.

We tested for collinearity between independent variables using the variance inflation factor (VIF), with defined problematic collinearity as a VIF greater than 10. No problematic collinearity was detected. We did not impute or otherwise include caregivers who provided data with missing responses for the measures included in our models. All analyses were conducted in R (version 3.4.1).

### Ethical Considerations

NHATS and NSOC were approved by the Johns Hopkins Institutional Review Board [24]. Our analyses were approved by the University of Alabama at Birmingham institutional review board (IRB) as Not Human Subjects Research (IRB #300011796).

## Results

### Participant Characteristics

We analyzed 463 responses representing 4,589,844 weighted family caregivers. Participants’ characteristics are summarized in Table 1.

**Table 1.** Participants’ characteristics.

Caregivers’ characteristics		Total sample, unweighted (N=463)	Caregivers of individuals with dementia (n=369)		Caregivers of individuals with cancer (n=104)	
		Value	Value	*P* value	Value	*P* value
Age in years, mean (SD)		63.4 (12.8)	63.4 (12.9)	.70	62.8 (13.1)	.13
**Race, n (%)**				.04		.27
	White	286 (64.6)	216 (60.8)		79 (78.2)	
	Black	126 (28.4)	109 (30.7)		18 (17.8)	
	Hispanic	31 (7)	30 (8.5)		4 (4)	
**Gender, n (%)**				.01		.51
	Male	157 (34.0)	128 (34.1)		34 (32.4)	
	Female	305 (66.0)	247 (65.9)		71 (67.6)	
**Employment status, n (%)**				.21		.23
	Employed	166 (37.4)	134 (37.3)		38 (38.4)	
	Unemployed	278 (62.6)	225 (62.7)		61 (61.6)	
**Health, n (%)**				.49		.83
	Good	230 (50.1)	181 (48.9)		60 (58.3)	
	Poor	229 (49.9)	189 (51.1)		43 (41.7)	
**Education level, n (%)**				<.001		.26
	College degree or higher	167 (40.7)	130 (39.6)		39 (42.9)	
	Lower than college degree	243 (59.3)	198 (60.4)		52 (57.1)	
**Religiosity, n (%)**				.39		.27
	Not religious	193 (43.6)	159 (44.5)		40 (40.8)	
	Religious	250 (56.4)	198 (55.5)		58 (59.2)	

### Statistical Results

Univariate statistical modeling results showed that, for caregivers of individuals with dementia, race (*P*=.04), gender (*P*=.006), and education level (*P*<.001) were associated with patient portal use. For caregivers of individuals with cancer, no covariates were associated with patient portal use (Table 1).

In the fully adjusted regression model for caregivers of individuals with dementia, identification with Hispanic ethnicity (OR: 2.81, 95% CI 1.05-7.57; *P*=.04) was associated with higher odds of patient portal use, whereas education lower than a college degree (OR 0.36, 95% CI 0.18-0.71; *P*<.001) was associated with lower odds of patient portal use. In the fully adjusted regression model for caregivers of individuals with cancer, no variables were statistically significantly associated with patient portal use at the .05 level. See Table 2 for more information on the fully adjusted regression modeling.

**Table 2.** Patient portal use by caregivers of individuals with dementia and cancer per fully adjusted regression modeling.

Predictors		Dementia			Cancer		
		Odds ratio	95% CI	*P* value	Odds ratio	95% CI	*P* value
Intercept		0.684	0.14-3.38	.64	0.16	0.01-3.5	.25
Caregiver age (years)		0.99	0.97-1.02	.54	1.01	0.96-1.05	.98
Female gender		1.49	0.71-3.14	.29	1.11	0.26-4.71	.89
**Caregiver race or ethnicity**							
	White (reference)	-	-	-	-	-	-
	Black or African American	0.64	0.30-1.34	.24	.34	0.06-1.94	.23
	Hispanic	2.81	1.05-7.57	*.04^a^*	0.98	0.12-8.15	.98
Unemployment		0.79	0.38-1.66	.54	1.64	0.41-6.59	.49
Poor caregiver health		1.16	0.59-2.27	.67	0.98	0.29-3.25	.97
**Education level**							
	College degree or higher (reference)	-	-	-	-	-	-
	Lower than a college degree	0.36	0.18-0.71	*<.01^a^*	1.44	0.41-5.06	.58
Religiosity		1.01	0.52-1.97	.98	1.88	0.57-6.12	.31

a Italicized values are statistically significant.

## Discussion

### Principal Results

Caregivers of individuals with cancer used the patient portal more than did caregivers of individuals with dementia. According to the regression modeling data, no caregiver demographics were associated with patient portal use among caregivers of individuals with cancer; however, among caregivers of individuals with dementia, Hispanic ethnicity was associated with higher odds of patient portal use, whereas education lower than a college degree was associated with lower odds of patient portal use.

### Comparison With Prior Literature

Our study expands on prior findings by Wolff et al [17] and Gleason et al [18], who identified the patient portal as an important method for engaging caregivers of patients living with dementia. Their studies conclude that family caregivers, especially those with high-need conditions, require additional informational support through the patient portal. Through a weighted analysis of a large national data set, our study examined the characteristics associated with portal use of two important groups of family caregivers who may benefit from future targeted intervention and support.

Our results underscore the need for tailoring approaches toward caregivers of individuals with cancer or dementia differently rather than using a one-size-fits all approach for interventions targeted at family caregivers. Differences between these caregiver groups have previously been identified as relevant to the caregiving experience, with caregivers for patients with cancer tending to provide care for a shorter duration, while caregivers of those living with dementia tending to provide care for comparatively longer periods [11,12]. A study comparing psychological distress in palliative care settings found caregivers of patients with dementia to have greater distress but similar burden, concluding that more work is needed to identify methods to further support family caregivers of patients with dementia [25]. Our findings highlight the need for tailored interventions that consider different patient conditions.

We found that caregivers of individuals with dementia who identify as Hispanic ethnicity had higher odds of using the patient portal, whereas caregivers of those with dementia with lower educational levels had relatively lower odds of using the patient portal.

In a study of 252 family caregivers who were provided access to physicians’ notes as part of a pre-post study, it was found that 87.5% of caregivers viewed these notes but that 46.7% did not know they were able to access the patient’s individual portal [14]. Caregivers’ reasons for viewing the physicians’ notes included seeking knowledge regarding the patient’s health (59.9%) and ensuring they understood what the doctor said (49.3%) [14]. Using the physicians’ notes was associated with improved communication with the patient’s health care provider [14]. While this study included patients aged 18 years and above and their caregivers with access to the patient portal account, the authors did not report on portal use categorized by condition or caregiver demographics. Our analysis expands on these findings by suggesting potential differences in informational needs based on caregivers’ demographic characteristics within different conditions, and these varied needs may influence the patient portal use by family caregivers. Indeed, there have been calls for a demographically diverse group of family caregivers to be engaged in the design of expanded electronic medical record systems [26]. Our findings highlight the importance of including Hispanic caregivers and those with lower educational attainment in the development of health record systems.

Studies involving Hispanic patients have found cultural and linguistic barriers to patient portal use. In one study, Hispanic patients were more likely to indicate that they did not require the patient portal for their own care [27]. The finding in our study that Hispanic family caregivers of individuals living with dementia had higher odds of using the patient portal could be attributed to cultural and linguistic factors. Hispanic cultures place high emphasis on familism [28-30]. Hispanic caregivers may have found the patient portal to be a valuable tool to assist them with their caregiving responsibilities.

When considering potential linguistic differences between caregivers and providers, especially when visits lack a certified medical interpreter, the patient portal could allow the caregiver to revisit information discussed during visits, facilitating a better understanding in the presence of linguistic differences. Nonetheless, information should ideally be communicated in the preferred language of the patient and caregiver [31]. This could be of particular importance for conditions like dementia and cancer, where visits are information-intensive, and recall can be negatively affected [32].

A study of 1996 caregivers through the 2011 NSOC found that Hispanic caregivers reported a higher percentage of unmet needs (49%) than did non-Hispanic caregivers (46.5%). Moreover, Hispanic caregivers had a much higher percentage reporting two or more unmet needs (42.6%) than did non-Hispanic caregivers (21.4%) [33]. Although this study does not examine the specifics of these informational needs, our findings suggest that these needs could be addressed by elements in the patient portal, such as direct messaging or validated informational documents. Future work examining what Hispanic caregivers find valuable in the patient portal, and what informational gaps the patient portal may or may not be addressing, is warranted and may have implications for other ethnic groups.

Other studies have also noted the differences in portal use among patients with lower educational attainment [34]. One barrier that has been identified is the ability to access the portal or comfortably use a computer [34]. As patient portals are often accessed online, internet access is a prerequisite, and comfort using technology is of high importance. About 25.9% of patients reported lack of comfort using a computer, although more work is needed to understand caregivers’ comfort using computers, especially as aging patients and caregivers may be becoming more familiar with computers [27]. A systematic review that sought to improve patient portal use by patients found that educational training sessions facilitated patient portal access by patients with lower educational attainment and lower levels of comfort using technology [35]. However, these interventions were also patient-focused and provided no data for family caregivers. Future work should include educational interventions for family caregivers.

Our analysis did not focus on palliative care specifically; however, we included conditions of individuals that commonly need palliative care as their conditions progress; specifically, about 35% of patients seeking palliative care do so for cancer or dementia [9].

### Limitations

Although our study is a weighted analysis of a methodologically strong, nationally representative data set, it is not without limitations. Comparatively few caregivers of cancer were included in the data set: only 22% (n=104) of caregivers in NSOC reported caring for individuals with cancer. As new, more complex treatments are developed and individuals with cancer are living longer with more advanced disease [33], it is important to target additional research to better understand and meet caregivers’ informational needs. Weighted analyses can make the sample representative by certain characteristics (in this case, age and race), but the weighting can artificially inflate the responses of the respondents being weighted, which can increase the sampling variance, standard error, and standard deviation. Although we adjusted for these characteristics in our regression model, the potential for sampling bias still exists. Additionally, we could not account for location in our sample, as those variables are part of a restricted file due to concerns of patient and caregiver privacy. Future research could consider the impact of rurality on patient portal use.

We also considered the limitations associated with multiple testing, but our study was exploratory in nature. While common adjustment methods such as the Bonferroni Correction or the Benjamini-Hochberg procedure can limit type 1 error rates, these methods can inflate type 2 error rates, which makes them less ideal for exploratory studies [36,37]. Previous studies have identified patient preference for in-person communication as an important factor when engaging with patient portals and health systems [38,39]. However, we did not have access to this information, and we were unable to include this as a factor in our analysis. Hence, future research should consider this important component that may influence caregiver behavior.

### Conclusion and Implications

Family caregivers often use the patient portal; however, we found differences in the patient portal use by different caregiver groups. We identified that caregivers of individuals with dementia use the patient portal at different rates, particularly those identifying as Hispanic and those with lower educational attainment. As the patient portal is a tool used by family caregivers to engage with health systems, health systems should consider cultural and educational interventions to support family caregivers with this critical aspect of their caregiving.

## Supplementary material

10.2196/44166 Checklist 1 RECORD (Reporting of Studies Conducted Using Observational Routinely-Collected Data) checklist.

## Abbreviations

IRB: institutional review board
NHATS: National Health and Aging Trends Study
NSOC: National Study of Caregiving
RECORD: Reporting of Studies Conducted Using Observational Routinely-Collected Data
VIF: variance inflation factor
